# Acceptability and solubility of iron and zinc contents of modified *Moringa oleifera* sauces consumed in the Far‐north region of Cameroon

**DOI:** 10.1002/fsn3.398

**Published:** 2016-06-21

**Authors:** Saliou Mawouma, Roger Ponka, Carl Moses Mbofung

**Affiliations:** ^1^ Laboratory of Biophysics, Food Biochemistry and NutritionDepartment of Food Science and NutritionThe University of NgaoundereNational School of Agro‐industrial SciencesPO BOX 455NgaoundereCameroon; ^2^ Department of Biological SciencesThe University of MarouaFaculty of SciencePO BOX 814MarouaCameroon; ^3^ Department of AgricultureLivestock and By‐ProductsThe University of MarouaThe Higher Institute of The SahelPO BOX 46MarouaCameroon

**Keywords:** Acceptability, acidulants, iron, *Moringa oleifera*, solubility, zinc

## Abstract

Consumption of *Moringa oleifera* leaves is a local and inexpensive solution to iron and zinc deficiencies in the Far‐north region of Cameroon. However, traditional household's cooking techniques result in sauces with high pH levels and low leaves incorporation rates that compromise the bioavailability of iron and zinc. The aim of our study was to investigate the effect of modifying a standard Moringa sauce on consumer acceptability and the solubility of iron and zinc, which is an indicator of their bioavailability. Lime juice or tamarind pulp was added to a standard recipe in order to reduce the pH by about one unit, and Moringa leaf powder was incorporated in each acidulated sauce at three levels (1, 2, and 4 g/100 g of sauce). All the formulations were evaluated for their acceptability by 30 housewives using a five‐point hedonic scale. The pH was measured by a digital electronic pH‐meter. Moisture and ash were determined by AOAC methods. Total iron and zinc contents were determined by atomic absorption spectrophotometry, and soluble iron and zinc by HCl‐extractability. The lime juice‐acidulated sauce and the tamarind pulp‐acidulated sauce enriched with 1 g of Moringa leaf powder were the most acceptable formulations with scores of 3.4 and 3.6, respectively. Their chemical analysis showed a reduced pH (6.4 and 6.1, respectively), compared to the Control (7.2). Lime juice‐acidulated sauce improved iron and zinc solubility from 42.19 to 66.38% and 54.03 to 82.03%, respectively. Tamarind pulp‐acidulated sauce enriched with 1 g of Moringa leaf powder showed a decrease in iron solubility from 42.19 to 38.26% and an increase in zinc solubility from 54.03 to 72.86%. These results confirm the beneficial effect of lime juice in improving iron and zinc bioavailability.

## Introduction

*Moringa oleifera* leaves are rich in iron and zinc, as well as vitamins A and C (Sena et al. 1998; Yang et al. 2006). These properties make these leaves an effective local and inexpensive solution in fighting iron and zinc deficiencies present in the Far‐north region of Cameroon (INS 2011). However, high contents of nutrients in food do not guarantee their effective usage in the body; these nutrients must be bioavailable (Watzke 1998). Recent studies have mainly focused on the nutritional quality of *Moringa oleifera* leaves, regardless of the complexity of the food matrix of recipes in which they are involved. Traditional cooking techniques are generally characterized by combinations of both activators and inhibitors of the bioavailability of trace elements. Our previous work on the characterization of traditional sauces made from *Moringa oleifera* leaves have revealed high pH levels and long cooking times at more than 100°C that compromise the bioavailability of iron and zinc. Also, we have found low leaves incorporation rates in sauces (not more than 17%), thus highlighting an under exploitation of the nutritional potential of these leaves (Mawouma et al. 2014). Organic acids promote the absorption of iron from plant foods by lowering the pH of food matrix and favoring the solubility of these minerals (Gillooly et al. 1983). Many studies have shown that addition of acidulants like tomato or tamarind juice played a role in increasing the available iron (Sathya et al. 2002; Hemalatha et al. 2005; Patted et al. 2013). Also, Wechtersbach and Cigić (2007)showed that lowering the pH of a solution containing dehydroascorbic acid allows its reduction into ascorbic acid, an enhancer of bioavailability of minerals. On the other hand, Moringa leaf powder, rich in iron, zinc, and vitamin A (recently shown to promote the solubility of iron), is used as a supplement to improve the nutritional quality of food in areas affected by malnutrition (Layrisse et al. 2000; Fuglie 2001; Dhakar et al. 2011). The aim of our study was to investigate the effect of adding two kinds of acidulant (lime juice or tamarind pulp) and Moringa leaf powder in a standard Moringa sauce on the consumer acceptability and the solubility of iron and zinc.

## Materials and Methods

### Formulation of samples

Fresh *Moringa oleifera* leaves were harvested from the same field located in the outskirt of Maroua. Part of these leaves were air‐dried under shade, milled into powder, sieved and stored in a well‐dried black plastic bag at room temperature of 25°C. The other ingredients were purchased from the main food market in Maroua town and stored at 4°C. The different samples were formulated as shown in Tables. The standard recipe (Control) were written based on information obtained from a field survey conducted in 243 households selected randomly from both rural and urban areas of the far‐north Region of Cameroon (Mawouma and Mbofung 2014). The modification of the Control consisted of: (1) adding two kinds of commonly used acidulants (lime juice or tamarind pulp) in order to reduce the pH of the Control by about 1 unit, that is 2.3 mL of lime juice/100 g of sauce and 2.9 g of tamarind pulp/100 g of sauce; (2) adding *Moringa oleifera* leaf powder to the previously acidulated sauces at three incorporation levels (1 g of powder/100 g of sauce, 2 g of powder/100 g of sauce, and 4 g of powder/100 g of sauce). The third level of incorporation (4 g of powder/100 g of sauce) was the maximum that did not drastically change the usual appearance of the sauces (viscosity and texture).

**Table 1:** Sample code and description of sauces acidulated with (A) lime juice and (B) tamarind pulp

Sample code	Description
(A) Lime juice	
S0	Control (Standard sauce):Fresh Moringa leaves (100 g), cottonseed oil (50 g), onion (45 g), peanut paste (70 g), tomato (70 g), salt (1.5 g), cube (4 g), tchoukouri (4 g), water (700 g).
S1L	Control + Lime juice (2.3 mL/100 g of sauce)
S2L	Control + Lime juice (2.3 mL/100 g of sauce) + Moringa leaf powder (1 g/100 g of sauce).
S3L	Control + Lime juice (2.3 mL/100 g of sauce) + Moringa leaf powder (2 g/100 g of sauce).
S4L	Control + Lime juice (2.3 mL/100 g of sauce) + Moringa leaf powder (4 g/100 g of sauce).
(B) Tamarind pulp	
S0	Control (Standard sauce):Fresh Moringa leaves (100 g), cottonseed oil (50 g), onion (45 g), peanut paste (70 g), tomato (70 g), salt (1.5 g), cube (4 g), tchoukouri (4 g), water (700 g).
S1T	Control + tamarind pulp (2.9 g/100 g of sauce).
S2T	Control + tamarind pulp (2.9 g/100 g of sauce) + Moringa leaf powder (1 g/100 g of sauce).
S3T	Control + tamarind pulp (2.9 g/100 g of sauce) + Moringa leaf powder (2 g/100 g of sauce).
S4T	Control + tamarind pulp (2.9 g/100 g of sauce) + Moringa leaf powder (4 g/100 g of sauce).

### Sensory evaluation

The products were subjected to sensory evaluation by a panel of 30 housewives who were regular consumers of *Moringa oleifera* sauces, using a five‐point hedonic scale ranging from 1 (dislike extremely) to 5 (like extremely). The evaluated parameters were texture, color, odor, taste and overall acceptability.

The most acceptable formulation for each kind of acidulants was selected for further analysis.

### Chemical analysis

The pH of the selected samples was recorded after homogenization by a domestic blender using a digital electronic pH meter (HANNA HI98103 Checker^®^ pH Tester, Cluj‐Napoca, ROMANIA). The moisture content was determined by drying fresh homogenized samples in an oven at 105°C until constant weight and ash by incineration in a muffle furnace at 550°C for 48 h (AOAC 1990).

### Determination of total iron and zinc

Minerals were determined according to the method described by Jones et al. (1990). The samples were ashed at 500°C for 10 h. The ash was then dissolved in 5 mL of deionized water and 15 mL of Aqua regia (a mixture of nitric acid HNO_3_ and hydrochloric acid HCl, in a molar ratio of 1:3). 1 mL of the obtained solution was diluted with 9 mL of deionized water. The minerals (iron and zinc) contents were determined using atomic Absorption Spectrophotometer (Buck Scientific 200 Serie AA, East Norwalk, Connecticut, USA.). All values were expressed in mg/100 g.

### Determination of iron and zinc solubility

After drying an aliquot of the homogenized wet samples at 42°C in an oven for 8 h, iron and zinc solubility was determined using the HCl‐extractability method described by Mahaja and Chauham (1988). One gram of each sample was extracted with 10 mL of HCl 0.03 N (the acid found in human stomach) by shaking at 37°C for 3 h. The clear extract obtained after filtration with a filter paper Whatman No. 42 was oven–dried at 100°C. The dried extract was then treated in the same way as the ash for determination of total iron and zinc. The solubility of each element was calculated using the following formula:

$$\text{Mineral solubility}\left(\%\right)=\frac{\text{Mineral soluble in HCl 0.03N}(\frac{\text{mg}}{100g})}{\text{Total mineral}(\frac{\text{mg}}{100g})}\times 100$$

### Statistical analysis

Data were presented as means ± standard deviation (SD). Values were statistically analyzed by one‐way analysis of variance (ANOVA test) using IBM SPSS Statistics version 20.0.1. Software package, Armonk, New York, USA.. Differences were considered significant at *P* < 0.05 using Duncan Multiple Range test.

## Results and Discussion

The sensory evaluation of the modified Moringa sauces is presented in Table 2. For all the parameters studied, the panel preferred the standard Moringa sauce (Control) over the modified sauces. The high score obtained for the Control as far as the overall acceptability is concerned (4.8) (*P* < 0.05) just confirm the good reputation of Moringa leaves as a vegetable food in the Far‐north region of Cameroon (Mawouma and Mbofung 2014). In details, the formulations S1L and S2T were the most generally acceptable ones (3.4 and 3.5, respectively) (*P* < 0.05). Odor and taste seemed to be crucial in the appreciation of the panel since they are the only two parameters that made the difference between the modified sauces.

**Table 2:** Sensory evaluation of samples acidulated with (A) lime juice and (B) tamarind pulp

Sample code	Texture	Color	Odor	Taste	Overall acceptability
(A) Lime juice					
S0	4.7^b ^± 0.6	4.8^b ^± 0.5	4.8^c ^± 0.5	4.8^c ^± 0.5	4.8^c ^± 0.5
S1L	3.4^a ^± 0.8	3.4^a ^± 0.8	3.4^ab ^± 0.8	3.4^b ^± 0.8	3.4^b ^± 0.8
S2L	2.9^a ^± 0.9	3.1^a ^± 0.9	2.9^a ^± 1.0	2.6^a ^± 1.0	2.7^a ^± 0.9
S3L	2.9^a ^± 1.0	3.0^a ^± 0.9	3.0^ab ^± 1.0	2.9^ab ^± 0.8	2.9^ab ^± 0.8
S4L	3.0^a ^± 1.1	3.0^a ^± 1.1	2.9^a ^± 1.1	2.6^a ^± 1.1	2.7^a ^± 1.1
(B) Tamarind pulp					
S0	4.7^b ^± 0.6	4.8^b ^± 0.5	4.8^c ^± 0.5	4.8^c ^± 0.5	4.8^c ^± 0.5
S1T	3.1^a ^± 0.7	3.1^a ^± 0.8	3.0^ab ^± 0.8	2.9^ab ^± 0.8	2.9^ab ^± 0.7
S2T	3.5^a ^± 0.8	3.5^a ^± 0.9	3.7^b ^± 0.7	3.6^b ^± 0.9	3.5^b ^± 0.9
S3T	3.3^a ^± 0.9	3.5^a ^± 0.9	3.5^ab ^± 0.8	3.1^ab ^± 0.9	3.2^ab ^± 0.9
S4T	3.1^a ^± 1.0	3.2^a ^± 1.0	3.2^ab ^± 1.1	2.9^ab ^± 1.2	2.9^ab ^± 1.1

Mean values in the same column with different superscript letters are significantly different (*P* < 0.05).

The result of the chemical analysis is presented in Table 3. The addition of Moringa leaf powder (1 g/100 g of sauce) to the tamarind pulp‐acidulated sauce did not significantly affect the reduced level of pH (*P* > 0.05). This observation is due to the neutral nature and the low quantity of the Moringa leaf powder that do not affect the pH of the sample to which it was added. No significant difference was noticed concerning moisture percentage (*P* > 0.05). The ash contents of the modified sauces were lower, compared to the Control (*P* < 0.05). The ash content is an indicator of the global mineral content of the samples. These results suggest that lime juice and tamarind pulp added were not rich in minerals and had a global reducing effect on the ash content of the Control. Although Moringa leaf powder is known to be rich in minerals (Moyo et al. 2013), the quantity added to the tamarind acidulated sauce (1 g of Moringa leaf powder/100 g of sauce) was too low to increase its ash content.

**Table 3:** Chemical composition of the most acceptable formulation of modified Moringa sauces for each acidulant

Sample code	pH	Moisture (%)	Ash (% of DM)
S0	7.2^b ^± 0.01	75.5^a ^± 0.6	8.2^b ^± 0.1
S1L	6.4^a ^± 0.01	75.1^a ^± 0.7	7.4^a ^± 0.3
S2T	6.1^a ^± 0.01	74.9^a ^± 1.5	7.9^a ^± 0.1

Mean values in the same column with different superscript letters are significantly different (*P* < 0.05). DM, Dry matter.

Table 4 indicates the influence of the modification of the Control sample on total and soluble iron (A) and zinc (B). No significant difference was observed for total iron and total zinc in Control and modified sauces (***P*** > 0.05). As previously said for ash contents, the low quantity of Moringa leaf powder added (1 g of powder/100 g of sauce for S2T) can explain this result. A similar fact concerning iron was obtained by Ndong et al. (2007) for a millet gruel enriched with <5% of Moringa leaf powder. This observation implies that there is a minimum amount of Moringa leaf powder to be added to a dish in order to improve its iron and zinc contents; the amount used in this study was not probably up to that threshold. It is advisable that the nutritional valorization of Moringa leaf powder in the Far‐north region of Cameroon should be done using other dishes where this leaf powder can be added in substantial quantities that can improve their nutritive value. The percentage of soluble iron and zinc was higher in the sauce acidulated with lime juice compared to the Control (iron, 66.38 and 42.19%, respectively; zinc, 82.03 and 54.03%, respectively). Citric acid found in lime juice is known to improve the bioavailability of minerals by increasing their solubility (Gillooly et al. 1983). A different situation was observed with the sauce acidulated with tamarind pulp where Moringa leaf powder was added: its iron solubility was lower (38.26%) than the Control (42.19%). This observation is comparable to the result obtained by Patted et al. (2013) after adding tamarind pulp as acidulant to an Indian vegetable dish (*Shepu dhalvada*). The chemical composition of tamarind pulp includes compound like polyphenols and phytates which are chelators that inhibit the solubility of minerals (Adeola and Aworh 2012). However, the inhibitory effect of those chelators affected only iron, since the zinc solubility was significantly higher in tamarind pulp‐acidulated sauce enriched with Moringa leaf powder (S2T), compared to the Control.

**Table 4:** Total and HCl‐extractable iron (A) and zinc (B) of the most acceptable formulation of modified Moringa sauces for each acidulant

Sample code	Total iron (mg/100 g DM)	Soluble iron (mg/100 g DM)	Soluble iron (% of total iron)
Iron (A)			
S0	16.71^a ^± 0.33	7.05^b ^± 0.01	42.19^b ^± 0.82
S1L	17.19^a ^± 0.70	11.41^c ^± 0.02	66.38^c ^± 2.60
S2T	16.99^a ^± 0.69	6.50^a ^± 0.24	38.26^a ^± 0.13
Zinc (B)			
S0	2.11^a ^± 0.04	1.14^a ^± 0.01	54.03^a ^± 0.57
S1L	2.17^a ^± 0.08	1.78^c ^± 0.01	82.03^c ^± 3.20
S2T	2.10^a ^± 0.03	1.53^b ^± 0.04	72.86^b ^± 2.66

Mean values in the same column with different superscript letters are significantly different (*P* < 0.05). DM, Dry matter.

## Conclusion

Modified Moringa sauces were developed by adding lime juice or tamarind pulp with Moringa leaf powder at three levels of incorporation to a standard recipe. The lime juice‐acidulated sauce and the tamarind pulp‐acidulated sauce enriched with 1 g of Moringa leaf powder/100 g of wet matter were the most acceptable formulations by a panel of 30 housewives. Their chemical analysis showed a reduced pH by about one unit compared to the standard sauce. Lime juice‐acidulated sauce improved iron and zinc solubility. Our study confirm the beneficial role of acidulants like lime juice in improving iron and zinc bioavailability.

## Conflict of Interest

The authors declare no conflict of interest.
